# Effects of yoga, aerobic, and stretching and toning exercises on cognition in adult cancer survivors: protocol of the STAY Fit pilot randomized controlled trial

**DOI:** 10.1186/s13063-020-04723-2

**Published:** 2020-09-15

**Authors:** Neha P. Gothe, Emily D. Erlenbach, Samuel L. Streeter, Linda Lehovec

**Affiliations:** 1 Department of Kinesiology and Community Health, University of Illinois at Urbana-Champaign, Urbana, IL 61801 USA; 2 Department of Dance, University of Illinois at Urbana-Champaign, Urbana, 61801 USA

**Keywords:** Yoga, Cognition, Fitness, Quality of life, Executive function

## Abstract

**Background:**

Cancer survivors experience compromised quality of life due to impaired cognitive function as a result of cancer diagnosis and treatment. Although exercise has proven to be effective in improving cognitive function across the lifespan, interventions comprehensively testing the effectiveness for cancer survivors are limited. The STAY Fit Trial is a three-armed pilot randomized controlled trial designed to compare the effects of a 12-week yoga, aerobic walking, and stretch and tone intervention on cognitive function among adult cancer survivors.

**Methods:**

This pilot study aims to recruit 75 adult cancer survivors who will complete assessments of cognitive function, cardiovascular fitness, physical activity, and psychosocial measures at baseline and after the 12-week intervention. The aims of STAY Fit are (1) to assess the efficacy of yoga to improve cognitive function among cancer survivors, compared to aerobic exercise and an active control group; (2) to examine changes in cardiovascular fitness as a result of the interventions; and (3) to assess changes in quality of life among our population as a result of the exercise interventions.

**Discussion:**

The STAY Fit Trial will test the effectiveness of yoga, aerobic exercise, and stretching and toning exercises in improving cognitive function and fitness among adult cancer survivors. The results of this pilot study will enable us to understand the most effective physical activity modality to improve cognitive function in this population and potentially combat cancer-related cognitive impairment.

**Trial registration:**

ClinicalTrials.gov NCT03650322. Registered on 28 August 2018.

## Background

Cancer is among the leading causes of death worldwide, yet scientific advances in prevention, diagnosis, and treatment have led to an increased number of cancer survivors estimated to be almost 19 million by 2024 [1]. However, survivorship does not come without an aftermath of physical health and quality of life complications. In 1998, the President’s Cancer Panel and the National Coalition for Cancer Survivorship formally recognized cancer-related cognitive impairment (CRCI) as a quality of life matter that deserved higher priority in clinical research [2]. CRCI is the loss of mental acuity associated with cancer and cancer treatment. About 75% of all cancer survivors report poorer attention and memory following treatment and the clinical prevalence of treatment-related cognitive impairment ranges from 17 to 75% [3, 4]. Cognitive processes thought to be impacted include impairment of memory, learning, concentration, reasoning, executive function, attention, and visual-spatial skills [5]. Despite the prevalence of cognitive impairment in cancer survivors, it is largely underdiagnosed and often left untreated [5, 6].

Although research on the beneficial effects of exercise training on cognitive functioning in non-clinical populations continues to emerge, parallel research examining cognitive functioning in cancer survivors is lagging. Among cancer survivors, many age-related physical and functional declines can be mitigated with exercise training [7]. In intervention studies, regardless of intervention specifics, exercise has been shown to improve quality of life and psychosocial and physical health outcomes in various cancer survivor groups during and after cancer treatment and may also help to manage long-term effects of treatment [8]. A majority of these interventions test aerobic exercise modalities, such as walking, as it is associated with improved health and cognitive function [9, 10]. However, few studies have examined the health benefits of yoga practice on cancer patients [11] and survivors [12].

Yoga is a noteworthy exercise modality that should be examined for its improvements in cognitive function and quality of life in clinical populations. As a holistic, mind-body activity, with components centered on meditation, breathing, and postures, it has shown great potential to improve cognitive function [13, 14]. Moreover, it is a low-impact exercise modality that may be more suitable by people limited in their mobility and function. However, little is known about the potential of yoga in maintaining or enhancing cognitive function and/or reducing CRCI among cancer survivors. An evidence-based review of yoga for cancer patients and survivors [15] reviewed 10 yoga-based interventions and noted some positive results, with outcomes primarily focused on psychosocial health and well-being (quality of life, depression, anxiety and stress being the most common). None of the reviewed interventions addressed cognitive function or CRCI.

STAY Fit is designed as a pilot three-arm randomized controlled trial, comparing the benefits of a 12-week yoga and aerobic walking intervention, using a stretching-toning group as an active control. In line with the purpose of conducting pilot studies, we designed this trial to test the feasibility of our methods and procedures for a future large-scale intervention and to explore possible effects and associations as a function of yoga practice on cognitive health [16]. The primary outcomes are measures of cognitive function, specifically domains of executive function, attention, memory, processing speed, and working memory. Secondary outcomes include cardiovascular fitness changes over the course of the intervention. Tertiary measures will examine changes in self-reported quality of life and psychosocial outcomes. Our protocol has been reported here in accordance with the SPIRIT checklist (see Additional file 1).

## Methods

### Study design

The STAY Fit trial is a three-arm randomized clinical exercise trial comparing the effects of a 12-week group-based yoga exercise, aerobic walking, and an active control (stretching and toning) on cognitive function and cardiovascular fitness among adult cancer survivors. We will randomize 75 low active adults between the ages of 30 and 70 years to one of the three conditions. All groups participate in structured group-based exercise led by certified and trained exercise leaders over the course of the 12-week intervention. Study outcomes include cognitive assessments, cardiovascular fitness, and psychosocial questionnaires administered at baseline and following the 12-week intervention.

### Duration of study

August 2018–May 2020.

### Subjects

The inclusion criteria are low physical activity engagement (i.e., less than 150 min of moderate to vigorous exercise per week), including no yoga practice over the last 6 months; aged between 30 and 70 years; capable of performing yoga, aerobic walking, or stretching and toning exercise without any pre-existing condition(s), as determined by their personal physician; the type of cancer (i.e., types of brain cancers are excluded as they could confound with cognitive study outcomes); the last treatment for cancer being at least 1 year from the beginning of the exercise program; not planning to receive any cancer-related treatment during the study period; intention to stay in the Champaign-Urbana area over the duration of the study; comfortable with reading, writing, and speaking English; and willingness to be randomized. Finally, all participants will be required to complete the Physical Activity Readiness Questionnaire (PAR-Q [17];) and must obtain approval from their personal physician, if deemed high-risk on the PAR-Q. The physician approval process will be facilitated by the research staff to minimize participant burden.

### Recruitment procedures

**Fig. 1:**
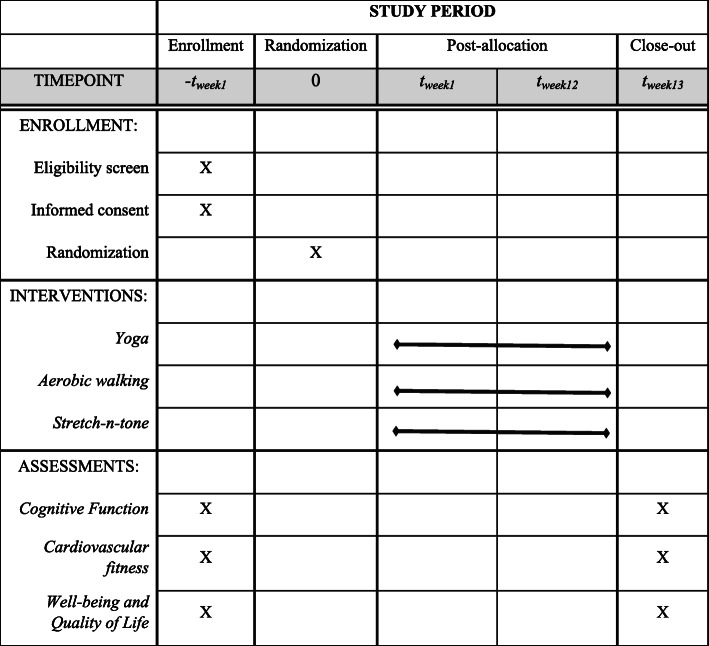
The STAY Fit Trial schedule of enrollment, interventions, and assessments

Our recruitment efforts include print, audio, and online media outlets to publicize the STAY Fit study in the Champaign-Urbana communities. All advertising materials include the key characteristics of the study—a free 12-week exercise program, opportunity to meet other cancer survivors from the community, monetary compensation for participation, and free parking. We will advertise in the local newspaper and place short ads on local radio, as well as attend various cancer survivorship and awareness events in the local area to distribute flyers and brochures to the attendees. Additionally, we will attend events at the local libraries and churches to distribute brochures. Finally, we will send an email or a letter to participants in the lab database who have been ineligible for previous studies with an invitation to participate in the new STAY Fit study (if deemed eligible). A schedule of enrollment, interventions, and assessments is presented in Fig. 1.

**Fig. 2:**
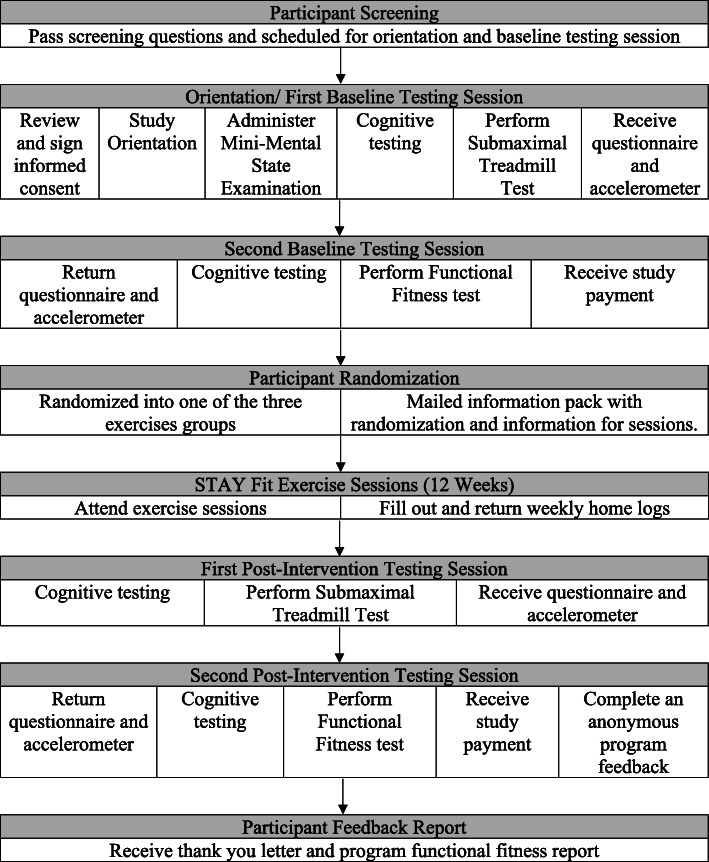
Flow of participants through the STAY Fit Trial

All participants who express interest will be provided with a description of the study and screened for eligibility. All participants will be required to read and sign a University Institutional Review Board-approved informed consent prior to study participation and data collection. Upon enrollment in the study, each participant will be assigned a random unique identification (ID) number as the only way to match collected data to their identifiable information. An electronic database, stored on a secure password-protected network only accessible by the study investigators, will house the key linking participant information and ID number. All written hard copies of participant data collected during the study period will only have the ID number written on them and will be stored in a secure location on the research site. Upon completion of the study, the key linking ID numbers and participant information will be destroyed. Participants will be informed that their de-identified data may be made available to regulatory authorities or ancillary individuals from the university, upon reasonable request. A snapshot of the flow of participants through the STAY Fit study is presented in Fig. 2.

### Randomization

Participants who are eligible for the study and complete all baseline measurements will be randomly assigned to 1 of the 3 intervention arms using a computer-generated program. Randomization will take place in groups following rounds of advertising and is stratified based on sex, age, and years since cancer diagnosis. Couples or friends who sign up for the study together will be randomized together to avoid contamination across groups. Randomization will take place following completion of all baseline assessments and a week before the 12-week exercise sessions are scheduled to begin. Participants will be notified via a mailed letter and follow-up phone calls confirming their randomized group and logistics (parking and directions) for their 12-week exercise sessions. This is an open label trial with no blinding of participants or assessors.

### Sample size calculation

We recognize that pilot studies do not typically report formal sample size calculations. However, to aid us in making advertising and recruitment decisions, we ran a very preliminary sample size analysis to get an estimated range of subjects needed. A meta-analysis of yoga effects on cognition [13] indicated an effect size range of .32–.47 (Hedge’s g) favoring the yoga condition. We based our sample size calculations to adequately power for a small effect within this range. Using an alpha of 0.05/2, a power of 0.80, and a conservative estimate of a medium effect size (*f* = 0.35), the desired total sample size is 75 (*N* = 25 per group) for this trial.

### Measures

All measures will be assessed at baseline and following the 12-week intervention. Measures of cognitive function (primary outcome) and cardiovascular function (secondary outcome) will be conducted in the laboratory by trained research assistants across two lab visits lasting approximately 2 h each. A battery of psychosocial questionnaires will be given to the participant to complete at home along with an Actigraph accelerometer (wGT3X-BT) that participants will be asked to wear for 7 days and nights to record objective levels of physical activity and sleep quality. We will schedule the testing appointments 1 week apart, so participants have time to complete the questionnaire packet and wear the accelerometer before returning to the lab.

#### Cognitive function (primary outcome)

The primary outcome of the trial is cognitive function. Specifically, we will measure executive function, attention and processing speed, memory, and working memory using the tests presented in Table 1. These measures have been used in the exercise-cognition literature and are partly adapted from the NIH Toolbox. The tasks are computer- and paper-based and will be administered over the two testing sessions on separate days and repeated again after the 12-week intervention. The first testing session consists of immediate and delayed word recall [18], flanker task [19], spatial working memory [20, 21], trail making [22], and matrix reasoning [23] task. The second session consists of relational memory [24], pattern comparison [25], and task switching paradigm [14, 21]. All measures involve a practice phase that explains the task instructions and allows participants to familiarize themselves with the test.

**Table 1:** Battery of neurocognitive tests administered at baseline and follow-up

Cognitive test	Cognitive domain	Approximate test duration
Word list (immediate and delayed recall)	Short- and long-term memory	5 min
Flanker task	Attention and inhibitory control	8 min
Spatial working memory	Spatial memory	15 min
Trail making A and B	Visual scanning, mental flexibility, processing speed	5 min
Matrices	Fluid intelligence	10 min
Spatial reconstruction task	Relational memory	Self-paced, 20 trials
Pattern comparison	Processing speed	4 min
Task switching	Mental flexibility	15 min

#### Cardiovascular function (secondary outcome)

The secondary outcome of the trial is cardiovascular function. A submaximal treadmill test will be administered at baseline and after the 12-week intervention. We will use a modified Balke protocol [26] that estimates a participant’s predicted VO2max based on the incline, speed, and heart rate over the last two stages completed during the test. The modified Balke is a well-accepted treadmill protocol that keeps the speed constant (3.0 mph) and workload by increasing the incline by 2.5% every 3 min. The test will terminate when the participant reaches 85% of their age-predicted maximum heart rate. We will then use their speed, incline, and heart rate from the last two completed stages, as well as their age-predicted maximum heart rate, to calculate their predicted VO2_max_ [27]. Before the test, anthropometric measurements including height, weight, resting heart rate, and resting blood pressure will be recorded. During the test, blood pressure and a rating of perceived exertion [26] will be recorded every 3 min for participant safety. Test will conclude once participant’s reach their 85% predicted maximum heart rate. In the event of participants taking prescription medications that are beta-blockers, a rating of perceived exertion of 17 will serve as an indicator for test termination. All participants will be led into a 3-min walking cool down at 2.0 mph followed by a 2-min seated rest. Heart rate and blood pressure will be monitored throughout the cool down to ensure participant is back at his/her resting state.

### Exercise intervention and active control groups

#### Yoga arm

The yoga intervention consists of two weekly, 90-min group sessions for 12 weeks. The style of yoga selected for the intervention is Hatha yoga, the most popular form of yoga practice in the USA [28] which focuses on practicing physical poses, breathing, and meditation. STAY Fit yoga sessions will be held at a local yoga studio and taught by a certified yoga instructor, who has been instructing and practicing for more than 20 years. Each 90-min session is structured such that participants perform 60-min of stretching and poses (*asana*), 15 min of guided breathing practice (*pranayama*), and 15 min of guided meditation. Table 2 depicts a sample yoga session. Sessions are designed at a beginner level for the first few weeks, with a focus on helping participants develop good form, proper alignment, and postures for the asanas. Props used during the sessions include a yoga mat, chair, blanket, and blocks.

**Table 2:** Sequence of Hatha yoga and stretching-toning exercises from week 1 of the STAY Fit Trial

Yoga	Stretching and toning
Poses (*asanas)*	Back arch
*Ardha Matsyendrasana*—twist in simple seated pose	Forward bend
*Marjaryasana/Bitilasana*—cat and cow	Squat
*Tadasana*—mountain pose	Military press
*Utkatasana—chair pose	Walking dips
*Urdhva Hastasasana*—upward salute	Bicep + fly arm cycle
*Virabhadrasana—warrior 1 and 2	Wood chop
*Purvottanasana—intense side stretch	Tricep extension
*Viparita Karani*—Supported legs on the seat of a chair	One leg stand
*Supta Baddha Konasana*—reclining bound angle pose	Side leg abductions
*Supported Savasana*—corpse pose	Bicycle abdominal twist
Breathing (*pranayama*)	
3 parts breath in *Supta Baddha Konasana*	
Mediation	
Guided meditation/body scan while in supported *savasana*	

Note: *Using chair for support

#### Aerobic walking intervention arm

Participants randomized to the walking intervention arm will meet three times a week, 60 min a session, for the 12-week intervention. The intensity of each walking session will be individualized for each participant. The 12-week structure of the walking sessions, including the intensity and duration for each week’s sessions, is presented in Table 3. The rating of perceived exertion (RPE) and target heart rate (HR) zones were selected in accordance with the ACSM exercise prescription guidelines for cancer survivors. Within each 4-week block, participants started at the lowest recommend intensity for 15 min, gradually increasing the duration to 40 min with the target HR zone. Prior to the start of the intervention, researchers will calculate each participant’s target HR zones using the Kavonen formula [29] ([(HR_max_ − HR_rest_) × % training intensity] + HR_rest_), where HRmax = 220 − age and HRrest = resting heart rate recorded at baseline submaximal treadmill test. All walking sessions are led by trained exercise leaders, who are trained with the detailed protocol for the sessions, and complete 2-h-long orientations to review the manual of procedures and protocols including participant safety, leading warm up and cool down exercises and adapting the intensity of exercise to target the HR zones. During each session, exercise leaders are responsible for monitoring the participant’s heart rate every 5 min to ensure they stay within their designated heart rate zone. Participants will be allowed to freely change the speed and/or incline during the session to stay in the target HR zone. If needed, participants will be given wrist and/or ankle weights to wear to keep them at a higher intensity. Sessions will begin with a 5-min warm up including dynamic stretching and brief aerobic activity (e.g., marching in place, high knees) and end with a 5-min slow walking cool down and static stretching until the participant HR returns to pre-exercise resting levels.

**Table 3:** ACSM’s aerobic exercise prescription for cancer survivors and protocol for the STAY Fit walking group

Weeks	1–4	5–8	9–12
Rating of perceived exertion	12–13	14–15	16–17
Target heart rate zone (% of heart rate reserve)	40–59	60–70	70–89
Duration (minutes)	15–40	15–40	15–40

#### Stretching and toning control arm

Participants randomized into the stretch and toning group will participate in sessions three times a week for 60 min each for the duration of the 12-week program. This group will participate in exercises that target all major muscle groups and work toward balance, toning, and flexibility. A sample stretching and toning exercise sequence is presented in Table 2. A given week will consist of the same exercises, but more reps and/or sets will be added over the course of the week’s sessions (Monday to Wednesday to Friday) building from 1 to 2 sets and 8 to 12 reps. Stretches and balance exercises were timed starting at 10 building up to 30 s. Exercises are designed to be progressive and challenging over the 12-week study period. At the beginning of each session, participants will walk around the perimeter of the room for 5 min and then engage in a 5-min warm up stretching routine. During the warm-up and before beginning the exercises, the leaders will inform the participants about the exercises, repetitions, and sets for the session. The exercise leaders will track the number of sets, repetitions, and time for exercises that are time-based (e.g., planks). Exercise leaders will provide posture and breathing cues throughout the session and demonstrate modifications for exercises when a participant presents mobility or functional limitations. The sessions will end with a 5-min cool down consisting of static stretching. Stretching and toning sessions will take place in an exercise studio and will be led by trained exercise leaders. Similar to the walking exercise leaders, these are given a manual detailing the protocol for the sessions and complete 2-h long orientation and training sessions to learn about participant safety, warm-up, stretching and toning exercises and modifications, and cool down stretches.

#### Exercise logs, attendance, and adherence

Prior to each session, participants in all groups will be fitted with a chest-strap heart rate monitor (Polar Model F1), which will record their heart rates over the session. At each session, participants will be given an exercise log, where they are to record their resting heart rate, average heart rate for the session, their self-reported rating of perceived exertion using the Borg scale [30], and self-reported enjoyment (a 1–5 Likert scale). The supervising exercise leader will keep track of the total minutes of exercise for the day and will review the logs for completeness before concluding the exercise session.

Attendance will be monitored by the respective exercise leaders for the 3 groups. A binder with attendance sheets for the 12 weeks will be presented to the exercise leaders at the first exercise session. Exercise leaders will be responsible for recording attendance and noting announced and unannounced absences. To promote adherence to the study protocols, participants will receive follow-up phone calls for any unannounced absences. They will also be given a mid-session feedback report (6 weeks, i.e., halfway into the intervention), providing averages of their weekly heart rates, rating of perceived exertion, enjoyment ratings, overall attendance percentage, and personalized feedback from their respective exercise leaders. Lastly, two times throughout the study period, exercise leaders will hold a raffle with prizes donated from local businesses (free fitness classes at a local gym) that can be redeemed after the trial ends.

In the event where a participant has decided to drop out of the study, their decision will be noted, and the participant will be contacted to administer follow-up assessments. Participants must have attended at least one session in order to be contacted for follow-up.

#### Home logs

Throughout the duration of the 12-week program, the participants will complete weekly home logs to track any participation in physical activity outside of the program. New home logs will be given to participants on Fridays and the completed home logs from the previous week will be collected by the exercise leaders on Mondays. Participants will be encouraged to record the type of physical activity they participated in, the intensity of the activity including an RPE rating and their enjoyment. If the participant does not engage in any home exercise, they will be asked to indicate “None” on the log.

### Adverse events and data safety monitoring

A data safety and monitoring plan has been established and approved by the University Institutional Review Board (IRB) that will allow us to consistently monitor and report adverse events to the University IRB. An official data safety and monitoring committee was not deemed necessary for this trial, as this is a single site, low-risk intervention. However, the investigators and research staff will meet on a weekly basis to review trial proceedings and participant status. All participants will be encouraged to report any health-related changes to the study staff. The exercise leaders will also closely monitor safety and follow up with participants in case of unannounced absences. If a participant discontinues exercise due to a health issue, the research staff will seek permission from their physician for resuming participating in the exercise program. If there is any recurrence of cancer or need for treatment (chemotherapy, radiation, or surgery) during the 12-week exercise sessions for a participant, he/she will be requested by the PI to discontinue participation in the trial as a safety and precautionary measure. Other than this, there will be no special criteria for discontinuing or modifying allocated interventions or stopping the trial.

### Statistical analysis

Data will be analyzed using SPSS 24.0 (IBM Corp., Armonk, NY). Each of the cognitive domains assessed will be analyzed separately. From each of the assessments administered, latent variables will be created to represent a global cognitive score for each domain. These variables will be created for both baseline and post-intervention test scores. Given the pilot nature of this study, we will focus on descriptive statistics and calculating effect sizes (Cohen’s d values). Data characteristics permitting (normality, multi-collinearity, sample size), we may conduct additional secondary analyses. For example, a time (baseline, follow up) × group (yoga, aerobic, stretching-toning) repeated measures analyses of covariance controlling for relevant covariates (possible covariates include age, education, stage of cancer, months since diagnosis, attendance, and baseline performance) can be employed to explore intervention effects for each of the cognitive domains. Similar repeated measures analyses could examine intervention effects for cardiovascular fitness and the tertiary psychosocial outcomes. Given that this is a pilot study, no additional subgroup or adjusted analyses are planned. An intent-to-treat analysis approach will be used for any dropouts who completed at least one exercise session. Missing data will be handled using a mean imputation approach. Participants’ exercise session attendance and physical activity performed outside of the session (as reported in home logs) will be factored into analyses as appropriate to control for participants’ adherence to study protocols.

## Discussion

### Strengths, limitations, and future considerations

To our knowledge, this is the first pilot randomized controlled trial to examine the effects of yoga on cognition in comparison to aerobic exercise and an active control group. Our use of a comprehensive neurocognitive battery will allow us to examine the global and selective effects of exercise on unique cognitive domains. However, this is a 12-week pilot trial and not without limitations. Although a number of yoga and aerobic exercise studies have demonstrated changes in cognitive function following 8- to 12-week interventions, longer interventions with multiple measurement time points can provide more evidence pertaining to the “dose-response” effect of exercise. The singular follow-up time point will limit us in assessing the maintenance effects for the study outcomes. Due to limited resources, our secondary outcome involves a submaximal fitness test to estimate cardiovascular fitness changes over the course of the intervention. Future studies could employ long-term follow-ups and a graded maximal exercise test that directly assesses peak VO_2_ capacity, which is considered the gold standard for cardiovascular measurement. We hope that our pilot study results will aid in informing a fully powered larger efficacy trial in the future. Results from our trial will also allow researchers to utilize more effective measures, perhaps use of magnetic resonance imaging and monitoring physiological data such as blood biomarkers, to investigate possible underlying mechanisms that mediate the exercise-cognition relationship.

## Conclusions

In addition to advancing the science of yoga and contributing to the fields of integrative medicine, aging, neurocognition, and cancer survivorship, the results of this proposed randomized control trial could have a significant impact on health care recommendations for cancer survivorship. Our study will determine the efficacy of yoga in improving cognitive health, and if proven effective, the gentle and amenable nature of yoga practice could have far-reaching applications for cancer survivors, many of whom are aging adults who will experience other age-related disabilities, co-morbidities, and overall compromised physical health. If found comparable to or better than aerobic exercise, yoga practice could be recommended and promoted as an alternative form of physical activity for adult cancer survivors to improve cognition and cancer-related cognitive impairment as well as physical and psychosocial health.

## Supplementary information

**Additional file 1.** SPIRIT checklist.

## Data Availability

This is a study protocol paper, and no datasets were used or analyzed in this manuscript. Requests for more details about the manual of procedures can be directed to the primary corresponding author. Any data required to support the protocol, such as the consent form and other related documentation given to participants, can be supplied on a reasonable request.

## Abbreviations

CRCI: Cancer-related cognitive impairment
PAR-Q: Physical Activity Readiness Questionnaire
RPE: Rating of perceived exertion
HR: Heart rate
